# Policy, Research and Residents’ Perspectives on Built Environments Implicated in Heart Disease: A Concept Mapping Approach

**DOI:** 10.3390/ijerph14020170

**Published:** 2017-02-09

**Authors:** Ivana Stankov, Natasha J. Howard, Mark Daniel, Margaret Cargo

**Affiliations:** 1 Urban Health Collaborative, Dornsife School of Public Health, Drexel University, Philadelphia, PA 19104, USA; 2 Centre for Population Health Research, School of Health Sciences, University of South Australia, Adelaide SA 5001, Australia; Natasha.Howard@unisa.edu.au (N.J.H.); Mark.Daniel@unisa.edu.au (M.D.); Margaret.Cargo@unisa.edu.au (M.C.); 3 South Australian Health and Medical Research Institute, Adelaide SA 5001, Australia

**Keywords:** built environment, cardiometabolic risk, concept mapping, community participation, policy, researcher perspectives

## Abstract

An underrepresentation of stakeholder perspectives within urban health research arguably limits our understanding of what is a multi-dimensional and complex relationship between the built environment and health. By engaging a wide range of stakeholders using a participatory concept mapping approach, this study aimed to achieve a more holistic and nuanced understanding of the built environments shaping disease risk, specifically cardiometabolic risk (CMR). Moreover, this study aimed to ascertain the importance and changeability of identified environments through government action. Through the concept mapping process, community members, researchers, government and non-government stakeholders collectively identified eleven clusters encompassing 102 built environmental domains related to CMR, a number of which are underrepresented within the literature. Among the identified built environments, open space, public transportation and pedestrian environments were highlighted as key targets for policy intervention. Whilst there was substantive convergence in stakeholder groups’ perspectives concerning the built environment and CMR, there were disparities in the level of importance government stakeholders and community members respectively assigned to pedestrian environments and street connectivity. These findings support the role of participatory methods in strengthening how urban health issues are understood and in affording novel insights into points of action for public health and policy intervention.

## 1. Introduction

Over the past decade, the increasing prevalence of cardiometabolic disease (CMD) [1,2,3], including type 2 diabetes mellitus and cardiovascular disease (CVD), has prompted substantial scientific enquiry. Much of this research [3] has focused on the identification of individual-level cardiometabolic risk (CMR) factors that predispose to the development and progression of related CMDs. Such risk factors include health-related behaviors, in particular those relating to diet and physical activity [3]. This mode of enquiry has translated only to limited success in addressing the global burden of CMD [4,5]. Those health benefits noted are, for the most part, short lived because individual-centered approaches tend to sideline important social determinants of inequities and inequalities in health [6,7]. Among these determinants is the residential context and its constituent characteristics. Given the growing consensus among health researchers concerning the salience of environmental factors [8,9] and the need for population-level interventions to address CMDs [10,11], attention has shifted to understanding the manner by which various facets of the built environment influence health outcomes, including CMR.

Within place-health research, the built environment, a dimension of the residential context, is defined as “that part of the physical environment made by people for people, including buildings, transportation systems, and open spaces” [12] (p. 558). A number of environmental features have shown a consistent association with CMR. In a systematic review of 131 studies dating back to 1986 [13], built characteristics, such as low residential density coupled with fewer street intersections, limited service availability and supermarket access, were all related to greater CMR (i.e., by virtue of CMR factors, such as obesity and hypertension). More recently, in 2013, a longitudinal study [14] found a significant association between land use mix, including density of recreational services, street network access and landscape slope, and BMI. In this body of research, built environments were conceived to influence the evolution of CMR primarily through their ability to enable or inhibit physical activity. These associations held after adjusting for a range of individual-level, socio-demographic and health-related factors. Beyond this, built environments may impact CMR through psychosocial and stress-axis pathways [15], as well as their innate capacity to shape the social context in which residents are embedded [16]. It follows then that inequitable access to built resources can give rise to population-level health inequities in CMR, through a range of pathways.

Research exploring associations between the built environment and health to date largely reflects the perspectives of researchers and their notions of place and disease [17]. Yet, the interrelationship between place and health is complex [18]. This complexity is attributable to interdependencies that exist between built environments, social and individual level factors (i.e., behavior, stress and psychosocial), whose reciprocal interactions function to shape the health and disease trajectories of populations. Given this complexity Harley and Fafard argue, that “researchers and the array of different decision-makers and community representatives with a stake in this issue may have widely divergent interpretations of both the problem and the salient solutions” [19] (p. 275). Though diverse stakeholder engagement is a central tenet of health promotion [20] and public health participatory research [21,22], the perspectives of residents and policymakers continue to be underrepresented within extant place-health inquiry [17,23]. The result is a fragmented understanding of the complex system of factors implicated in the development of CMR and the actions that may curb their incidence.

It is contended that the mismatch between current conceptualizations of place-health dynamics and the complexity from which they arise can effect policy resistance [18]. As characterized by Sterman, this resistance is a “tendency for interventions to be delayed, diluted, or defeated by the response of the system to the intervention itself” [24] (p. 5), a phenomenon evidenced both within health policy [25] and population-level research [26,27]. Through engagement with residents, researchers and policymakers, this study aimed to better understand the place-health system through an explication of built facets considered both relevant to CMD and amenable to change through government action.

Concept mapping (CM), a participatory mixed-method approach widely used in the social sciences, is a methodological tool that has gained traction within place-health research [28,29,30,31] and public health [32] due to its ability to enable the development of conceptual frameworks reflective of diverse stakeholder perspectives. Using this approach, this study aimed to address the following objectives within an urban Australian context: (1) to formalize a concept map that represents facets of the built environment influential in shaping heart health, from the perspective of researchers, policymakers and community members; (2) to determine the overall relative importance and changeability of built features identified in Objective 1; and (3) to compare these ratings within and between stakeholder groups.

## 2. Study Context

This study was supported by the National Health and Medical Research Council funded Place and Metabolic Syndrome (PAMS) Project (GNT631917) [33] and Partnership (GNT570150) [34] grants. The PAMS Project aimed to understand the associations between neighborhood-level attributes and CMR, using individual-level biomedical data and area-level environmental data from the North West Adelaide Health Study. The Partnership component, which was co-funded by the South Australian (SA) Department for Health and Ageing, sought to facilitate knowledge translation of analytic outcomes concerning place-health relations through collaboration between researchers and partnering organizations from government and non-government sectors. The PAMS Advisory Group was comprised of community, not-for-profit and government partners who provided strategic input into the direction of the Project and the translation of knowledge into public health action. Underpinned by a university-community partnership, the PAMS Project provided a stakeholder network and the infrastructure to support this CM study.

The PAMS Project was aligned with the SA Health in All Policies (HiAP) Initiative [35], which recognized the need for cross-sectoral collaboration in the development of health-centered public policies. The SA Public Health Act 2011 [36] further demonstrated the Government’s commitment to the principles of the HiAP Initiative. Within The Act, state and local governments were tasked with legislative responsibility to develop a State and Local Public Health Plan, which, among other aims, sought to support and improve the health of communities and neighborhoods. The aims and objectives of the CM study were therefore well placed in supporting policymakers across a range of government sectors, to fulfil the mandates of the SA Public Health Act 2011.

Under the rubric of the PAMS Project, the CM study focused on the northwest Adelaide region, South Australia. At baseline in 2001, this region represented 38% of the Adelaide metropolitan region and 28% of the total state population [37]. Though this region encompasses residents of diverse socio-demographic strata, the proportion of low income families is higher (28%) than in the remaining metropolitan areas (19%) [38]. Moreover, the prevalence of CMR factors, including high blood pressure (32.7%) and cholesterol (37.3%), as well as overweight and obesity (72.4%), is particularly high within the northwest Adelaide region [39,40,41]. In efforts to address the issues affecting the region, the SA Government outlined priorities aligned with place-health inquiry, among them the establishment of partnerships across government sectors and settings [42].

## 3. Methods

Concept mapping [43,44], or structured conceptualization, is a participatory approach which uses both qualitative processes and quantitative analyses to integrate stakeholders’ perspectives on a given topic of interest [45]. The use of CM is particularly pertinent in policy analysis, management and public health, where often ill-defined and complex human interactions are at stake [46]. Its reliability has been established across a range of projects [47]. The CM approach [48] consists of six steps; preparation, brainstorming, sorting and rating, analysis, the interpretation of maps and knowledge utilization.

### 3.1. Recruitment

Three participant groups were approached to capture a range of perspectives related to features of the built environment. These included individuals: (1) conducting research with a place-health focus; (2) residing in the northwest region of Adelaide; and (3) from government and non-government organizations (i.e., policymakers). These participant groups will be referred to hereafter as researchers, community members and “(non)government” stakeholders, respectively. A uniquely-targeted recruitment strategy was employed for each group.

(Non)government stakeholders concerned with urban planning and/or health were recruited using purposive sampling. Potential participants were identified through established investigator networks. A snowball sampling strategy was subsequently employed to identify other individuals working for relevant organizations. The aim of this approach was to maximize sample diversity, across two dimensions: (1) portfolio (e.g., urban planning, health); and (2) seniority of role (e.g., professor, research fellow, project officer, manager). Snowball sampling was also adopted to enable the identification of volunteer and community groups with established links to (non)government organizations, from which community members could be recruited. This approach was complemented with the display of pamphlets at a local library and health service. Those recruited were asked to identify other eligible residents.

Individuals in the community member sample were screened for inclusion based on a minimum two-year residence in the northwest Adelaide region. Furthermore, participant selection aimed to achieve diversity in the sample based on: (1) age (under 35, 35–55, 55+); and (2) residential representation across Local Government Areas within the northwest region (*n* = 5).

Researchers from the three SA universities were purposively sampled. Prospective participants were screened against the following eligibility criterion; must have previously conducted research with a place-health focus (i.e., exploring feature(s) of the built and/or social environment in relation to health or wellbeing) as evidenced by at least one peer-reviewed publication.

Interested individuals from each of the three groups were sent an information sheet via email and were followed-up after one week. Informed consent was attained from all participants prior to involvement in the study. The study was conducted in accordance with the Declaration of Helsinki, and the protocol was approved by both the University of South Australia and the SA Department for Health and Ageing Human Research Ethics Committees (P030-10 and HREC/13/SAH/108, respectively). All data were collected between 18 November 2013 and 17 January 2014.

### 3.2. Data Collection Procedure

#### 3.2.1. Preparation

The focus statement was pilot tested on three individuals, one from each stakeholder group, to determine its effectiveness and clarity in eliciting information relevant to the aim. Following participant feedback, amendments were made to the focus prompt and instructions featured within the Concept System Global MAX (Version 4.0) software [49]. These changes related primarily to language, in particular the adoption of the term “heart health” in place of “cardiometabolic risk”; the former was perceived to be more familiar to a range of stakeholders. Once formalized, all recruited participants were sent a web-based link to the online software. Participants were required to create a profile using an email address or username. They were then prompted to complete a series of demographic questions relating to gender, ancestry, educational attainment and relationship to the northwest Adelaide region. All three activities, namely, brainstorming, rating and sorting, were conducted on the online platform.

#### 3.2.2. Brainstorming

Participants (*n* = 43) were asked to brainstorm ideas and concepts in response to the focus statement: “A characteristic of the built environment, in the northwest Adelaide region that could influence, in any way, directly or indirectly, a person’s heart health is...” Participants were prompted to think broadly about the issue and to brainstorm statements that directly or indirectly explain how they perceive the place-health relationship. Following completion of this phase, statements were pooled and discussed by a working group comprising members of the research team (Ivana Stankov, Margaret Cargo, Natasha J. Howard) as well as two community members and a (non)government stakeholder. During this process, identical statements were consolidated, while very descriptive ideas were integrated and renamed under broader headings. The final list of statements was subsequently used in the sorting and rating phases of the study.

#### 3.2.3. Rating

Forty seven participants were invited to rate the final set of statements. During this activity those partaking were instructed to rate all statements on a 5-point scale against two dimensions: relative importance and changeability through government action within the next five years. The prompts used to facilitate this process included: importance: “Relative to others, to what extent do you perceive each of the following statements to be important in influencing the heart health of adults living in the northwest Adelaide region. Please rate on a scale of 1–5”; changeability: “Relative to others, to what extent do you perceive each of the following statements to be changeable through government intervention, within the next five years. Please rate on a scale of 1–5”.

#### 3.2.4. Sorting

All participants (*n* = 47) were asked to complete the sorting exercise. As part of this process, participants were instructed to create piles based on the similarity of statement constructs. This grouping could occur in any fashion, provided that three rules were followed. First, statements could not all be placed in the same pile; second, each statement could not form a pile on its own; and third, dissimilar statements could not be grouped together in one pile. Finally, participants were required to provide a suitable label describing each pile.

### 3.3. Analysis

The Concept System Global MAX software [49] was used to conduct the CM analysis. A unique binary matrix of similarities$\;\left(X_{N\times N}\right)$, with N representing the total number of statements, was generated for each participant using the information gained from the sorting activity. For any given cell $X_{ij}$, where i and j each represented a given pair of statements, a “1” was entered if the statements were placed in the same pile and a “0” entered if this was not so [50]. This process was repeated for each participant involved in the sorting activity. Cells across$\;X_{N\times N}$ matrices were thereafter summed to produce another N×N similarity matrix$\;\left(T_{N\times N}\right)$ with each cell containing an integer value between 0 and P. Here, P denotes the total number of participants involved in the sorting process, while the value in each cell $T_{ij}$ represents the total number of people that placed items i and j in the same pile during sorting. The total matrix $T_{N\times N}$ was analyzed using nonmetric multidimensional scaling (MDS) analysis [51], which, as suggested by Kruskal and Wish, uniquely represents each statement by means of an x and y coordinate [52]. The resulting bivariate plot of coordinates provides a visual representation of the relative similarity of statements based on their proximity to each other. A similarity cut-off value of “1” was used to ensure that statements grouped in the same pile by only one participant were excluded from the modelling process. This level of filtering was imposed to reduce potential “noise”, which may be created by spurious statement placement likely to arise given the ambiguity inherent in the subject matter under investigation. Therefore, statements depicted visually as closest together on the map are those that were grouped together most frequently by participants during the sorting process, and vice versa, those furthest apart were grouped together least frequently.

A stress value between zero and one was calculated to indicate the goodness of fit of statement configurations, where a smaller value is indicative of a smaller discrepancy between the bi-dimensional representation of the data and the input similarity matrix$\;\left(T_{N\times N}\right)$. Values between 0.205 and 0.365 represent a good fit for field-based projects [52]. Furthermore, bridging values generated during the MDS analysis, ranging from zero to one, were used to assess the extent to which each statement is “anchored” to a particular place on the map [48]. A statement that is an “anchor” (i.e., characterized by a low bridging value), for example, is one that reflects well the content in its vicinity.

The (x,y) coordinates resulting from the MDS were used as input for hierarchical cluster analysis [53,54]. This procedure divided the statement map into non-overlapping clusters using Ward’s algorithm [53,55]. The software assigned a label to each cluster on the point-cluster map based on participant labels provided during the sorting phase. Following the completion of the concept map, a bivariate comparison of the average cluster ratings on perceived importance and changeability was conducted, in the form of a “ladder” graph, to elucidate aggregate patterns [56]. Confidence intervals (CI; 95%) were determined around ratings of mean importance and changeability, which allowed for cross-group comparisons by cluster. Furthermore, importance and changeability relationships were also assessed at the statement level using a “go-zone” plot, where the (x,y) coordinate of each statement was plotted in bivariate space, the quadrants of which were determined by the mean x and y values. The “go-zone” plot is named after the top, right-hand quadrant, which depicts the statements of both greatest relative importance and changeability.

### 3.4. Interpretation of Maps

Following the completion of data analysis, the working group re-convened to assist with the interpretation of the final concept map. During this process, group members reviewed cluster labels assigned by the software, discussed whether these appropriately described the meaning of statements contained within and renamed the clusters as necessary. Further discussion focused on the location of statements within clusters and specifically, whether statements ought to be moved from one cluster to another or if the division or merger of clusters was warranted. Subsequent to a number of refinements, the final concept map was approved by all members of the working group.

## 4. Results

Forty seven individuals completed at least one of the CM activities. Forty three participants completed the brainstorming phase: 18 researchers; 13 (non)government stakeholders; and 12 community members. Participant representation across (non)government stakeholder, researcher and community member groups followed an approximate ratio of 1:1.5:1.5, respectively, across both sorting (*n* = 32) and rating (*n* = 34) phases. The attrition rate varied by activity with far lower rates of attrition observed for the brainstorming activity (8.5%) relative to the rating (27.7%) and sorting (31.9%) phases. Table 1 provides demographic details for completers, by activity.

### 4.1. Sorting

**Figure 1:**
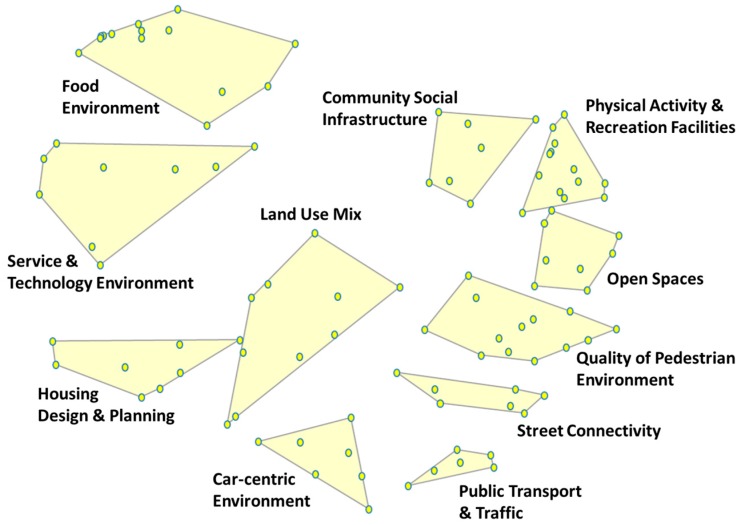
Concept map of 11 built environments capable of shaping cardiometabolic risk (CMR).

An 11-cluster concept map represents all statements sorted by researchers (*n* = 12), (non)government stakeholders (*n* = 8) and community members (*n* = 12). The concept map (Figure 1) had a stress value of 0.25, denoting a good fit between the sorting data and cluster configurations [51]. The final cluster map reflects consensus between working group members from all three stakeholder groups, with respect to both cluster labels and statement groupings.

The majority of cluster bridging values (presented within Supplementary Materials Table S1) ranged between 0.32 and 0.65. This represents an overall moderate level of cluster anchoring to their respective regions on the map. The “Food Environment” and “Service and Technology Environment” clusters are comprised of statements with both the lowest and highest bridging values, respectively. That is, the “Service and Technology Environment” cluster, with a relatively high bridging value (0.81), is a product of statements that were frequently grouped with items other than those in their immediate vicinity. As such, the function of this cluster is to bridge other regions of the map. Conversely, statements comprising the “Food Environment” were grouped together most frequently during the sorting process and are therefore relatively firmly “anchored” to their position on the map. Also of note is the proximity of statements that comprise the top left boundary of the “Food Environment” cluster. These statements are conceptually more closely related to each other than to those on the bottom right margin of the same cluster.

### 4.2. Rating

**Figure 2:**
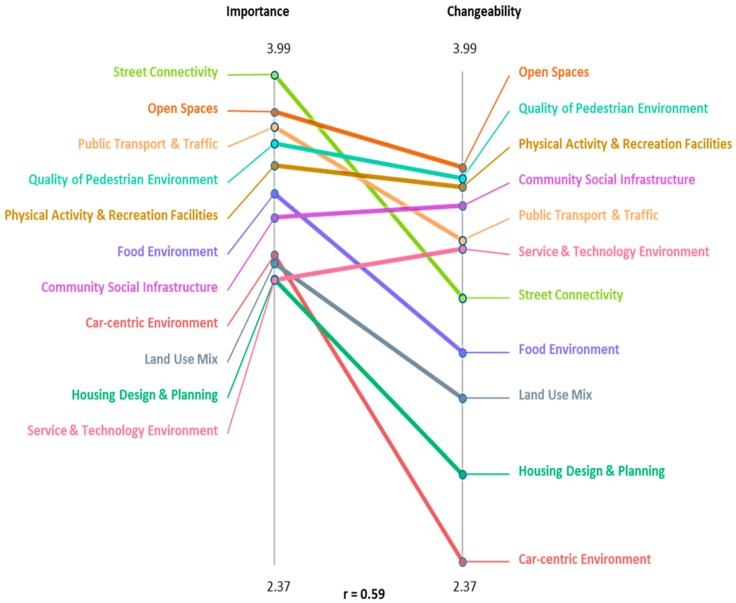
Ladder graph depicting the relationship between average importance and changeability ratings, for all participants, by cluster.

The mean importance and changeability ratings, across all participants (*n* = 34), at the statement and cluster levels, are detailed in Supplementary Materials Table S1. Information depicted at the cluster level, within Figure 2, highlights those built environmental domains rated as very important, but receiving comparatively low scores on the changeability dimension. Characterized by steep negative slopes, these domains include “Street Connectivity”, “Food Environment”, “Car-centric Environment”, “Land Use Mix” and “Housing Design and Planning”. Of particular note is the “Street Connectivity” cluster rated as most important, but ranked seventh on the changeability scale. Conversely, clusters with more concordant importance and changeability ratings, such as “Open Spaces” and “Quality of Pedestrian Environments”, were identifiable by flatter line gradients. The correlation between relative importance and changeability at the cluster level (r = 0.59) was greater than at the statement level as indicated by the Pearson product-moment correlation.

**Figure 3:**
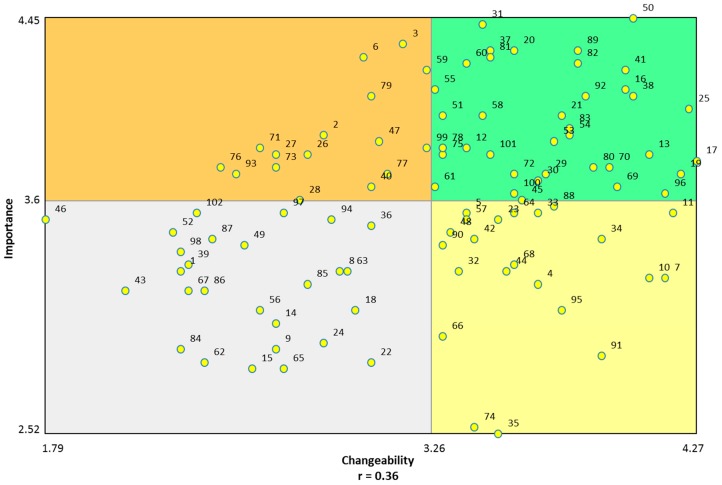
“Go-zone” of statement changeability and importance, for all participants.

Importance and changeability ratings were used to construct a “go-zone” plot (Figure 3), wherein the top, right-hand quadrant depicts statements of greatest relative importance and changeability. Of all 102 brainstormed statements, 37 were located within the “go-zone”. Of the 11 clusters, eight clusters included statements represented within the “go-zone” (demarcated with an asterisk in Supplementary Materials Table S1). The three clusters not represented were “Car-centric environments”, “Housing Design and Planning” and “Land Use Mix”. The overall correlation between relative importance and changeability ratings of statements was modest (r = 0.36).

To account for between cluster differences in the number of statements comprising each cluster and the influence of these differences on the appearance of statements within the “go-zone”, an additional calculation was performed. This calculation of relative representation was derived by dividing, for any given cluster, the number of its statements appearing in the “go-zone” (demarcated with an asterisk in Supplementary Materials Table S1) by the total number of statements comprising that cluster. Through this process, clusters identified as having the highest relative representation of statements within the “go-zone” were: “Open Spaces” (88%), the “Quality of Pedestrian Environments” (77%); and “Public Transport and Traffic” (67%). As featured in Supplementary Materials Table S1, top rated statements with respect to their combined average importance and changeability, within each of these clusters included: “Child-friendly environments to encourage families to use urban space” (ID#82); “The provision of useable bike paths or lanes for recreation” (ID#16); and “Accessible public transport to encourage mobility, reduce isolation and encourage walking” (ID#37).

#### Differences by Participant Group

**Figure 4:**
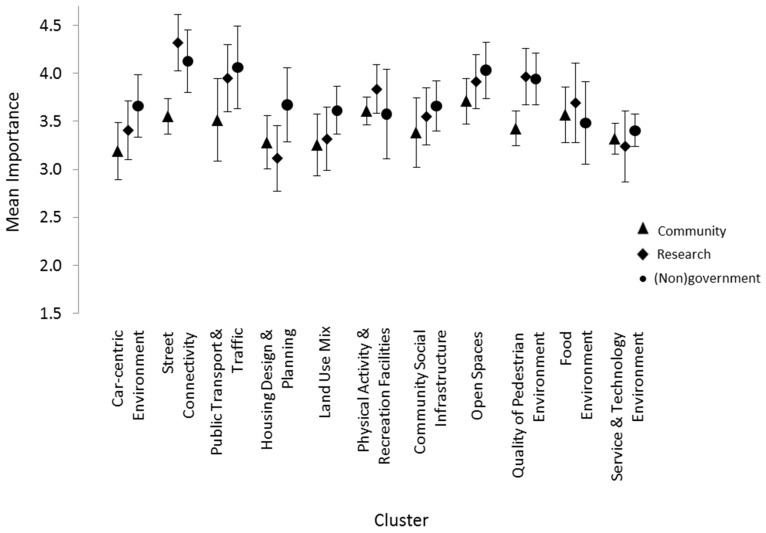
Mean importance ratings (with 95% confidence intervals) according to cluster, by participant group.

**Figure 5:**
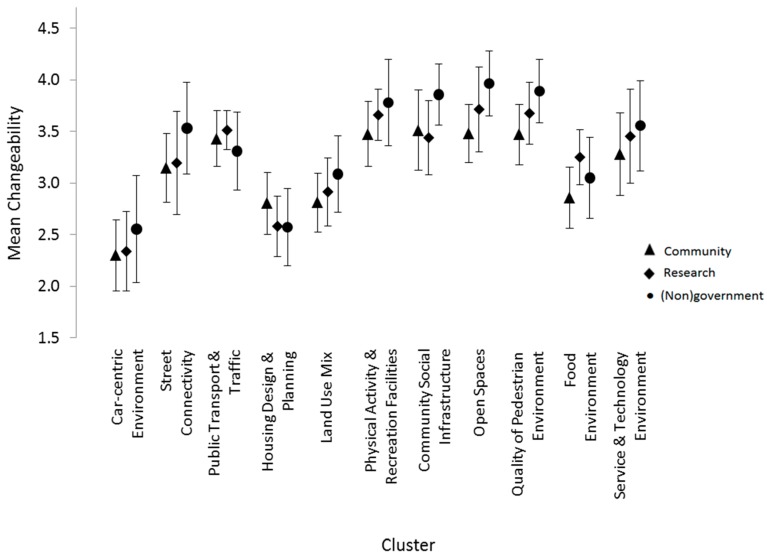
Mean changeability ratings (95% confidence intervals) according to cluster, by participant group.

Each rating variable was assessed independently, and comparisons were made across the three stakeholder groups for each cluster. With respect to mean importance ratings, community members and researchers, as well as researcher and (non)government stakeholder groups were closely correlated (r = 0.75 and 0.76, respectively). There was, however, greater divergence in the mean importance ratings between the community member and (non)government stakeholder pair (r = 0.44) across all clusters. Confidence intervals (95%) constructed around each mean importance rating (Figure 4) enabled across-group comparisons, by cluster. Such contrasts revealed non-overlap between community group ratings relative to the other two stakeholder groups for Clusters 2 (“Street Connectivity”) and 9 (“Quality of Pedestrian Environment”). No stakeholder group differences were found for mean cluster changeability ratings (Figure 5). This was in keeping with the high cluster correlation for changeability ratings observed across all groups, i.e., community and researchers (r = 0.94), researchers and (non)government stakeholders (r = 0.92) and community and (non)government (r = 0.91) groups. Simply, these findings suggest that stakeholders had similar perspectives concerning the changeability of built environmental clusters.

## 5. Discussion

This study represents a critical first step in efforts to integrate researchers’, (non)government stakeholders’ and community members’ mental models of the built environment and its relationship to heart health. Of the 11 environmental clusters that emerged as influential constructs, from the perspective of stakeholders, five environments feature prominently within contemporary place-health research focused on CMR. Specifically, “Food Environments”, “Public Open Spaces”, “Physical Activity and Recreation Facilities”, “Street Connectivity” and “Land Use Mix”. The remaining six clusters, however, including, “Car-centric Environments”, “Public Transport and Traffic”, “Community Social Infrastructure”, “Quality of Pedestrian Environment”, “Housing Design and Planning” and “Service and Technology Environment”, represent environmental domains that, to date, have received little attention within the CMR literature. At the statement level, the findings of this work suggest potentially important and changeable policy and intervention foci. Moreover, the results of this study highlight important differences in stakeholder perceptions of the role built environments play in shaping heart health. These findings are further discussed below in the context of study strengths and limitations.

The CM approach resulted in the emergence of 11 environmental domains. Of these, several are normatively highlighted in the place-health literature concerned with CMR. Specifically, “Food Environments” (the focus of a large portion of investigations [57,58,59,60]), “Public Open Spaces” [61,62], “Physical Activity and Recreation Facilities” [57], “Street Connectivity” and “Land Use Mix” [63,64]. The remaining environmental domains featured within the cluster map, however, have to-date received little or no attention in the scientific literature concerned with CMR [13]. These include “Car-centric Environments”, “Public Transport and Traffic”, “Community Social Infrastructure”, “Quality of Pedestrian Environment”, “Housing Design and Planning” and the “Service and Technology Environment”. Interestingly, two of these environmental domains (i.e., “Quality of Pedestrian Environments”, ranked fourth in importance and second in changeability, and “Public Transport and Traffic”, ranked third in importance and fifth in changeability within the ladder plot) were identified as having among the highest relative representation of statements within the “go-zone”. This finding signals a mismatch between the perceived importance and changeability of these environments, and the attention they have thus far received within place-health inquiry focused on CMR.

Studies that have explored how the “Quality of Pedestrian Environments” and “Public Transport and Traffic” shape health outcomes did so by limiting their focus to health-related behaviors, such as physical activity [65]. Relationships between these two environments and CMR have, to date, been under investigated and remain less well understood. Research focused on investigating the “Quality of Pedestrian Environments” observed negative associations between sidewalk buffers (i.e., fences, trees and grass) and blood pressure [66]. Another found street-level attributes of the “Public Transport and Traffic” system, including traffic control measures (i.e., traffic signals, speed bumps and stop signs) to be inversely correlated with resting heart rate [66]. Whilst the relationships identified in these studies are aligned with expectations, a number of studies have reported findings inconsistent with expectations. These include positive associations between (i) pedestrian/bicycling environment attractiveness and time spent in motorized transport [66] and (ii) sidewalk completeness and BMI [67]. Furthermore, the observed direction of associations between subway/bus stop density and BMI has been found to vary across ethnic groups [67]. These observed inconsistencies may in part be attributable to methodological limitations or variations in approaches used across studies. Likewise, they may reflect the complexity of the mechanisms that underpin these place-health relationships.

“Open Spaces”, “Public Transport and Traffic” and “Quality of Pedestrian Environments”, as environments with the greatest relative representation of statements within the “go-zone”, represent important and changeable targets for intervention. At the statement level, identified as particularly influential and changeable within the “Open Spaces” cluster, were ideas emphasizing child-friendly environments in determining the use of open spaces by families (i.e., ID#82) and the presence of trees providing shade and shelter in public spaces (i.e., ID#19). In the context of “Public Transport and Traffic”, accessible and reliable public transport (i.e., ID#37 and #81) was outlined as particularly influential in shaping heart health, while the provision of usable bike paths or lanes for recreation (i.e., ID#16) and strategically placed trees and bushes (i.e., ID#17) were identified as important features related to the “Quality of Pedestrian Environments”. The ideas embodied within these statements suggest the types of intervention strategies that may be most influential and actionable within the region.

The results additionally highlight important differences in stakeholder group perceptions concerning the role of built environments in shaping heart health. For instance, study findings revealed that all stakeholder groups agreed on the changeability of each built environmental domain represented within the concept map. Cluster-level importance ratings across community member and (non)government stakeholder groups, however, were poorly correlated. This disagreement related specifically to the perceived importance of “Street Connectivity” and the “Quality of Pedestrian Environments” in shaping heart health. On average, community members deemed both of these environmental domains to be of lower importance relative to the other stakeholder groups. This difference in perspective may reflect two important underlying issues. The first relates to potential stakeholder disparities, in their respective beliefs, concerning the capacity of environments to induce health-enabling shifts in people’s behavior [63]. The second points towards a disconnect in the appreciation that (non)government stakeholders have of the priorities and values of resident populations. In the context of the northwest Adelaide region, this difference in perspectives highlights the need for continued community-level consultations to inform public health planning and legislation [42].

On the whole, the final concept map represents a good fit between the sorting data and point distances between statements [47], indicated by a stress value of 0.249. High bridging values however, were observed for most items, with the exception of a small group of statements found within the “Food Environment” cluster. This finding denotes a relatively high degree of variation in the sorting of items into their constituent clusters, suggesting diversity in stakeholders’ mental models. Underlying explanations for this particular result could be interpretive or conceptual, depending on a given stakeholder. Beyond the language used in framing dimensions of place, notions concerning the similarity of constructs could have contributed to the observed variation. Furthermore, it is possible that sorting instructions were interpreted differently by participants.

Notable strengths of this study are its use of a university-community platform and its alignment with the SA HiAP initiative [68] and the SA Public Health Act 2011 [36]. By building on established partnerships with local and state government departments with a legislative responsibility to improve population-level health and wellbeing, the findings of this work may be used to support the formalization and implementation of state-wide interventions and policies. For instance, the identification of public transit and traffic environments as among the most important and changeable determinants of CMR lends support to transportation-focused efforts, including the provision of a high quality transportation system, proposed within both the 30-year Plan for Greater Adelaide [69] and the Integrated Transport and Land Use Plan for the region [70]. As participants directly involved in the development of this knowledge, policymakers are well placed to advocate for and support policies aligned with the outcomes of this study.

The findings of this research should be interpreted in light of a few limitations. The first relates to the use of purposive sampling during the recruitment phase to maximize sample size and diversity and thereby facilitate the representation of disparate perspectives. Whilst sampling strategies aimed to maximize participant numbers and heterogeneity across stakeholder characteristics, the final sample did not fully reflect these efforts. For example, men were underrepresented across all activities while (non)government stakeholders were outnumbered by the other two groups in both rating and sorting phases. This has implications for the generalizability of findings. In particular, through the process of averaging across stakeholders within groups, the cluster-level importance and changeability pattern matching graph may inadequately capture the perspectives of (non)government stakeholders. Moreover, stakeholders from other sectors with potentially novel insights into the place-health interrelationship may not have been captured by the employed sampling strategy. The overall sample size, albeit small relative to other web-based CM studies, is within the range of studies conducted using this methodology [47]. The generalizability of study findings to other settings is also limited by the specificity of the focus prompt, which sought to identify environmental constructs specific to the northwest metropolitan region of Adelaide.

These limitations aside, the findings of this study suggest a need for further research investigating associations between built environments, particularly public transportation/traffic systems and pedestrian environments, and CMR. Additionally highlighted is the importance of integrating diverse stakeholder perspectives, using participatory approaches such as CM, in efforts to understand the system of interrelated factors implicated in the development of CMDs. Methodological tools drawn from the systems sciences may enable a deeper exploration of, and insights into, these place-health dynamics [71,72,73]. Applied to the design of CMD interventions, an understanding of the system within which CMDs emerge could minimize instances of policy resistance and thereby optimize population-level health outcomes. This is particularly pertinent given that many governments across the globe are seeking to enact policies and programs intended to enrich residential environs through action directed toward their built features.

## 6. Conclusions

Supported by a university-community partnership and the engagement of local stakeholders, this study aimed to better understand features of the built environment influential in shaping heart health in an urban setting. Public “Open Spaces”, “Quality of Pedestrian Environments” and the “Public Transit and Traffic” environment were identified by stakeholders as being among the most important and changeable determinants of CMR, according to their relative representation within the “go-zone”. Important and changeable targets for action within each of these domains, as identified by the most highly rated statements, include the provision of: child-friendly urban spaces that are appealing to families; usable bike paths for recreation; and an accessible public transportation system that encourages mobility and reduces isolation. The outcomes of this study also highlighted marked differences between the perspectives of (non)government stakeholders and community residents concerning the importance of two environs: “Street Connectivity” and the “Quality of Pedestrian Environments”. More broadly, the findings of this research contribute to a more holistic conception of the place-health system relative to existing models derived primarily from the researcher perspective. Future research utilizing participatory methodologies should aim to extend these observations by focusing specifically on open space, public transit and traffic and quality dimensions of pedestrian environments.

## Figures and Tables

**Table 1:** Demographic summary of participants by concept mapping activity.

Categories	Brainstorming (*n* = 43)		Sorting (*n* = 32)		Rating (*n* = 34)	
Stakeholder type						
Researcher	18	(41.9%)	12	(37.5%)	14	(41.2%)
(Non)government stakeholder	13	(30.2%)	8	(25.0%)	8	(23.5%)
Community member	12	(27.9%)	12	(37.5%)	12	(35.3%)
Gender						
Female	23	(53.5%)	21	(65.6%)	23	(67.7%)
Male	12	(27.9%)	10	(31.3%)	10	(29.4%)
No response	8	(18.6%)	1	(3.1%)	1	(2.9%)
Ancestry						
United Kingdom	12	(27.9%)	10	(31.3%)	10	(29.4%)
Australian	16	(37.2%)	13	(40.6%)	14	(41.2%)
European	2	(4.7%)	3	(9.4%)	3	(8.8%)
Other	5	(11.6%)	5	(15.6%)	6	(17.7%)
No response	8	(18.6%)	1	(3.1%)	1	(2.9%)
Highest qualification attained						
Postgraduate degree	17	(39.5%)	16	(50%)	18	(52.9%)
Bachelor degree	11	(25.6%)	12	(37.5%)	12	(35.3%)
Vocational education	3	(7.0%)	2	(6.3%)	2	(5.9%)
Year 12 or below	2	(4.7%)	1	(3.1%)	1	(2.9%)
Other	2	(4.7%)	0		0	
No response	8	(18.6%)	1	(3.1%)	1	(2.9%)

## Supplementary Materials

The following are available online at www.mdpi.com/1660-4601/14/2/170/s1. Table S1. Statements with accompanying bridging values, mean importance and changeability ratings and combined average importance and changeability ratings, by cluster.
