# Supplementary material for: Policy, Research and Residents’ Perspectives on Built Environments Implicated in Heart Disease: A Concept Mapping Approach
Source: Int J Environ Res Public Health. 2017 Feb 9;14(2):170. doi: 10.3390/ijerph14020170 (PMC5334724; doi:10.3390/ijerph14020170)
Supplement: Supplementary file 1 [file ijerph-14-00170-s001.pdf]

Supplementary Materials: Policy, Research and Residents' Perspectives on Built Environments Implicated in Heart Disease: A Concept Mapping Approach

Ivana Stankov, Natasha J. Howard, Mark Daniel and Margaret Cargo

Table S1. Statements with accompanying bridging values, mean importance and changeability ratings and combined average importance and changeability ratings, by cluster.

ID	Statement	BV	I	C	Avg I and C
Cluster 1: Open Spaces	Relative representation of statements in the go-zone (88%)	0.33	3.87	3.68	3.78
82	* Child-friendly environments to encourage families to use urban space	0.31	4.24	3.82	4.03
19	* The presence of trees providing shade and shelter in public spaces	0.28	3.73	4.21	3.97
70	* The presence of dog parks that are close by to walk to and meet people	0.36	3.76	3.94	3.85
58	* Public spaces that are 'visible' spaces; spaces such as parks which are visible from the street and by neighboring residents, promoting natural surveillance and improved safety	0.36	4.00	3.45	3.73
7	The presence of leash-free dog walking facilities	0.37	3.24	4.15	3.70
55	* The amount of nice gardens, street trees and parks in the area for clean air, outdoor activities and exercise	0.30	4.12	3.27	3.70
51	* The visibility of open spaces, sporting grounds and other activity centers from major roads	0.34	4.00	3.3	3.65
78	*Open space both public and private	0.31	3.85	3.3	3.58
Cluster 2: Quality of Pedestrian Environment	Relative representation of statements in the go-zone (77%)	0.34	3.76	3.65	3.71
16	* The provision of useable bike paths or lanes for recreation	0.44	4.12	4.00	4.06
17	* Trees and bushes planted strategically to cool and soften the physical environment	0.28	3.79	4.27	4.03
13	* Adequate lighting to generate feelings of safety and management of vegetation/overgrowth to inhibit issues of concealment	0.29	3.82	4.09	3.96
96	* Street lighting	0.29	3.64	4.15	3.90
21	* Quality footpaths that are set a reasonable distance from the road side, away from cars	0.30	4.00	3.76	3.88
69	* Rundown or damaged public open space facilities	0.36	3.67	3.97	3.82
80	* Beach paths with way-finding signs and interesting art pieces	0.32	3.76	3.88	3.82
60	* Quality open spaces connected to paths and other local destinations	0.31	4.24	3.39	3.82
53	*Tree-lined streets and footpaths on both sides of the street	0.28	3.88	3.73	3.81
101	* A pleasant local area to walk in	0.43	3.82	3.48	3.65
79	The presence of destinations to walk to, or local features to walk past from home or school	0.39	4.09	3.03	3.56
63	Street level activity	0.38	3.27	2.94	3.11
65	The presence of underground versus above-ground services (power, telephone, etc.) influencing how attractive the local environment is for exercise	0.33	2.82	2.70	2.76

Table S1. *Cont.*

Cluster 3: Public Transport and Traffic		Relative representation of statements in the go-zone (67%)	0.33	3.82	3.44	3.63
37	* Accessible public transport to encourage mobility, reduce isolation and encourage walking		0.34	4.30	3.48	3.89
81	*Access to reliable public transport		0.36	4.27	3.48	3.88
72	* Safe speed environments created by reducing and calming vehicles to facilitate safe pedestrian and cyclist movement along residential and collector streets		0.29	3.73	3.58	3.66
61	*Poor public transport connectivity across the region		0.37	3.67	3.27	3.47
57	Public transport access to walking beaches and Torrens Linear Park		0.32	3.52	3.39	3.46
42	Traffic calming measures		0.30	3.42	3.42	3.42
Cluster 4: Street Connectivity		Relative representation of statements in the go-zone (43%)	0.34	3.99	3.25	3.62
89	* Continuous and safe bike corridors (i.e., bike lanes do not disappear into traffic)		0.32	4.30	3.82	4.06
92	* Clear, safe and separated bike paths leading to the city and local schools		0.32	4.09	3.85	3.97
59	The walkability of the local area		0.35	4.21	3.24	3.73
75	* Pathways and connecting links between cul-de-sacs		0.31	3.82	3.30	3.56
47	The presence of walking paths that have under/over passes over main arterial roads (i.e., to encourage safe walking)		0.33	3.88	3.06	3.47
26	Commercial destinations within walking or cycling distance from homes		0.37	3.82	2.79	3.31
27	High street connectivity (grid-type street pattern)		0.37	3.82	2.67	3.25
Cluster 5: Community Social Infrastructure		Relative representation of statements in the go-zone (43%)	0.44	3.52	3.55	3.54
29	*Outdoor areas with organized programs to encourage interaction and exercise with others		0.41	3.73	3.70	3.72
30	* The provision of social neighborhood hubs and meeting places such as corner stores, cafes and libraries		0.42	3.70	3.67	3.69
88	Community centers where there are activities to participate in		0.52	3.58	3.73	3.66
45	* A high number of community centers in the region, with emphasis on exercise for elderly programs		0.41	3.61	3.61	3.61
23	Places to gather or meet friends		0.45	3.52	3.52	3.52
91	The presence of community and public artwork to document history and culture and create colorful textures and images		0.37	2.88	3.91	3.40
28	The presence of gardens which mean that people get out and exercise while weeding, watering and maintaining the garden		0.48	3.61	2.76	3.19
Cluster 6: Physical Activity and Recreation Facilities		Relative representation of statements in the go-zone (38%)	0.33	3.69	3.62	3.66
50	* The availability of public open space with supportive facilities (e.g., toilets, water fountains, pathways, BBQ area, picnic tables, rubbish bins, car parking adjacent to parks)		0.33	4.45	4.03	4.24
25	* The availability of playground equipment for children		0.31	4.03	4.24	4.14
41	*Access to well-maintained parks and playgrounds (i.e., that feel safe for parents to bring children) with facilities for a range of ages		0.33	4.21	4.00	4.11
38	* Community access to school sports grounds and facilities		0.31	4.09	4.03	4.06
83	* Access to local, well-lit ovals (i.e., for evening sporting activities)		0.31	3.94	3.79	3.87
10	Well promoted "come and try" days at local sports venues, especially encouraging adults to play sports		0.42	3.24	4.09	3.67
34	Council-provided playgrounds and activities, designed by "risk averse" culture, that are boring for kids and therefore used rarely		0.38	3.42	3.91	3.67
5	The presence of a central hub with facilities for a variety of sporting activities and wellbeing		0.32	3.55	3.39	3.47
44	The presence of outdoor gyms		0.31	3.27	3.55	3.41

Table S1. *Cont.*

77	Access to safe, inclusive and affordable local sports clubs	0.36	3.73	3.09	3.41
40	The availability of local swimming pools	0.3	3.67	3.03	3.35
8	Access to gyms	0.34	3.27	2.91	3.09
18	The availability of local gyms	0.32	3.09	2.97	3.03
Cluster 7: Food Environment	Relative representation of statements in the go-zone (29%)	0.24	3.60	3.06	3.33
20	* The provision and access to healthy food	0.17	4.30	3.58	3.94
31	* Access to, and affordability of, fresh produce	0.03	4.42	3.45	3.94
54	* Communal places to grow fresh foods, including school vegetable gardens, shared orchards, verge planting and small scale urban food producers	0.69	3.91	3.79	3.85
3	The saturation of unhealthy food outlets	0.01	4.33	3.15	3.74
6	The build-up of fast food outlets, on popular routes and near low income housing	0.00	4.27	3.00	3.64
12	* The amount of junk food advertising	0.12	3.85	3.39	3.62
64	Farmers Markets which enable local produce to be sold for healthy eating	0.14	3.55	3.58	3.57
93	Accessibility of fast food outlets	0.02	3.73	2.52	3.13
74	Recycled organic waste which benefits soil fertility	0.64	2.55	3.42	2.99
22	The attractiveness of local supermarkets	0.63	2.85	3.03	2.94
24	The number of pubs/clubs in the local area (i.e., access to alcohol)	0.66	2.94	2.85	2.90
52	The ease of buying or having junk food delivered to your door	0.02	3.45	2.27	2.86
98	The presence of backyard vegetable gardens for food	0.15	3.36	2.30	2.83
15	A large selection of Asian restaurants and shops which offer an alternate healthy option over general western diet	0.11	2.82	2.58	2.70
Cluster 8: Service and Technology Environment	Relative representation of statements in the go-zone (11%)	0.81	3.31	3.41	3.36
11	Access to schools for education on healthy lifestyles	0.69	3.55	4.18	3.87
100	* Access to health services	0.74	3.64	3.58	3.61
33	A workplace providing health promotion programs	0.82	3.55	3.67	3.61
4	Education opportunities	0.74	3.21	3.67	3.44
95	The location of smoking areas outside of buildings	0.73	3.09	3.76	3.43
48	Appropriateness of health services	0.77	3.45	3.33	3.39
66	Access to new technologies in order to enable information seeking	0.89	2.97	3.30	3.14
49	Employment opportunities	0.92	3.39	2.55	2.97
9	The increased use of online technology and virtual service providers that meet people's needs without having to leave the home	1.00	2.91	2.67	2.79
Cluster 9: Land Use Mix	Relative representation of statements in the go-zone (0%)	0.49	3.36	2.91	3.14
68	The identification and transformation of underutilized land and public spaces, particularly along the railway line	0.41	3.3	3.58	3.44
2	Low dwelling density, poor land use mix (i.e., nothing to walk too) and concentrated retail opportunity	0.49	3.91	2.85	3.38

Table S1. *Cont.*

90	The integration of new developments and older/historical dwellings to reduce feelings of isolation and separation, promote daily mobility, create sense of vibrancy and dynamic change	0.47	3.39	3.30	3.35
36	Medium density housing, located in activity centers, with plenty of amenities	0.44	3.48	3.03	3.26
71	The quality of the air	0.56	3.85	2.61	3.23
76	The centralization of core services (i.e., butchers, bakeries and deli's) into large shopping centers, rather than smaller district or local service areas/centers which encourage people to drive to do weekly shop rather than walking to do small/frequent shopping trips	0.62	3.76	2.45	3.11
35	Graffiti	0.61	2.52	3.52	3.02
85	Built environments that reflect dignity, culture and self-determination	0.45	3.21	2.79	3.00
14	Single building allotments, leading to isolation within the neighborhood as people become more fearful and less likely to go outside as they age	0.46	3.03	2.67	2.85
67	Fewer industries in suburban areas	0.42	3.18	2.33	2.76
Cluster 10: Housing Design and Planning	Relative representation of statements in the go-zone (0%)	0.59	3.31	2.66	2.99
32	Public housing in poor condition	0.54	3.27	3.36	3.32
73	Poorly designed neighborhoods with housing features that reduce community interactivity	0.62	3.76	2.67	3.22
94	The density of public housing within the local area (which could influence economic status, social cohesion, crime, safety, etc.)	0.50	3.52	2.88	3.20
97	The size of housing and allotments influencing the green space available in backyards for exercise (e.g., gardening and backyard cricket)	0.56	3.55	2.70	3.13
102	High house prices and general living costs force people to work too much which prevents people (younger generations in particular)from having an active and healthy lifestyle	0.70	3.55	2.36	2.96
56	Type of access in multi-level buildings (i.e., option to take the stairs rather than the lift)	0.65	3.09	2.61	2.85
62	Housing tenure	0.62	2.85	2.39	2.62
84	Housing style	0.55	2.91	2.30	2.61
Cluster 11: Car-centric Environment	Relative representation of statements in the go-zone (0%)	0.42	3.39	2.37	2.88
99	The nature of new residential developments, with emphasis on non-linear roads (and huge number of cul-de-sac suburbs) resulting in the need for car transport for basic shopping trips	0.42	3.85	3.24	3.55
87	Car and industry centric environments	0.42	3.42	2.42	2.92
39	The volume of motor vehicles on the road	0.36	3.30	2.33	2.82
1	Industries estates too far (or too inconveniently) from residential areas, making it difficult for workers to travel in ways other than by car	0.45	3.27	2.30	2.79
86	The placement of shopping center car parks at the back or underground which improves ease of access to pedestrians	0.48	3.18	2.39	2.79
46	The distance needed to travel to work	0.47	3.52	1.79	2.66
43	Major roads through suburbs	0.35	3.18	2.09	2.64

BV = bridging value; I = importance rating; C = changeability rating; Avg I and C = combined average importance and changeability ratings; * = statements in the go-zone.

© 2017 by the authors. Submitted for possible open access publication under the terms and conditions of the Creative Commons Attribution (CC BY) license (http://creativecommons.org/licenses/by/4.0/).
